# Improve the Performance of Soy Protein-Based Adhesives by a Polyurethane Elastomer

**DOI:** 10.3390/polym10091016

**Published:** 2018-09-13

**Authors:** Yecheng Xu, Yantao Xu, Wenjie Zhu, Wei Zhang, Qiang Gao, Jianzhang Li

**Affiliations:** 1 Key Laboratory of Wood Material Science and Utilization, Beijing Forestry University, Beijing 100083, China; xuyecheng@bjfu.edu.cn (Y.X.); xuyantao@bjfu.edu.cn (Y.X.); zhu1012524632@163.com (W.Z.); zhangweishe@126.com (W.Z.); 2 Ministry of Education, Beijing Key Laboratory of Wood Science and Engineering, College of Materials Science and Technology, Beijing Forestry University, Beijing 100083, China

**Keywords:** soy protein, polyurethane elastomer, interpenetrating network, water resistance, bonding strength, joined crosslinking network

## Abstract

The purpose of this study was to improve the performance of soy protein isolate (SPI) adhesives using a polyurethane elastomer. Triglycidylamine (TGA), SPI, thermoplastic polyurethane elastomer (TPU), and γ-(2,3-epoxypropoxy) propyltrimethoxysilane (KH-560) were used to develop a novel SPI-based adhesive. The residual rate, functional groups, thermal stability, and fracture surface micrographs of the cured adhesives were characterized. Three-ply plywood was fabricated, and the dry/wet shear strength was determined. The experimental results suggested that introducing 2% TGA improved the residual rate of the SPI/TGA adhesive by 4.1% because of the chemical cross-linking reaction between epoxy groups and protein molecules. Incorporating 7% TPU into the SPI/TGA adhesive, the residual rate of the adhesive increased by 5.2% and the dry/wet shear strength of plywood bonded by SPI/TGA/TPU adhesive increased by 10.7%/67.7%, respectively, compared with that of SPI/TGA adhesive. When using KH-560 and TPU together, the residual rate of the adhesive improved by 0.9% compared with that of SPI/TGA/TPU adhesive. The dry and wet shear strength of the plywood bonded by the SPI/TGA/TPU/KG-560 adhesive further increased by 23.2% and 23.6% respectively when compared with that of SPI/TGA/TPU adhesive. TPU physically combined with the SPI/TGA adhesive to form a interpenetration network and KH-560 acted as a bridge to connect TPU and SPI/TGA to form a joined crosslinking network, which improved the thermo stability/toughness of the adhesive and created a uniform ductile fracture section of the adhesive.

## 1. Introduction

In recent years, soy protein-based adhesives (SPIAs) have garnered great research attention as a substitute for formaldehyde-based adhesives, to eliminate formaldehyde emission from wood-based panels [1]. As the most important raw material of SPIAs, soybean meal is abundant, inexpensive, renewable, biodegradable, and nontoxic [2,3]. But the practical application of SPIAs has been limited, due to the low water resistance [4]. Chemical and physical methods were applied to enhance water resistance of the SPIAs, including denaturation [5], cross-linking agents [6], synthetic resin [7], nano-material [8] and biomimetic modification [9]. Among those modifications, the most effective way is using cross-linking agents and synthetic resins, such as phenol formaldehyde resin [10], polyisocynates [11], polyamidoamine–epichlorohydrin resin [12] and epoxide [13]. The active functions of the cross-linker or synthetic resin react with the –NH_2_, –NH– and –COOH groups of the protein to generate a cross-linked structure, which improves the water resistance of the adhesive. However, our previous research showed that the SPIAs are brittle, and this brittleness of the adhesive further increases after modification by a cross-linker, resulting in low dry bond strength and impact resistance properties of the bonded panel. This is because the residual stresses within the panel are inevitable from the manufacture process, and these residual stresses will increase with the moisture content increase/decrease in the panel during the using process. These residual stresses are usually balanced by the bond force of cured adhesive; however, if the cured adhesive is brittle, this balance will be easily broken when the residual stresses increasing. Previous research has shown that increasing the cross-linking agent dosage, although forming a more compact structure of the adhesive, reduces the bond strength of the resultant panel [14], which was in accordance with the analysis above. Therefore, increasing the toughness of the SPIAs will benefit for balancing the interior forces, which improves the bond strength and water resistance of the adhesive.

In this study, soy protein isolate (SPI) and a laboratory-synthesized crosslinker (triglycidylamine, TGA) were used to develop a SPI-based adhesive. In order to improve the toughness of the adhesive, a thermoplastic polyurethane elastomer (TPU) and γ-(2,3-epoxypropoxy) propyltrimethoxysilane (KH-560) were additionally incorporated to develop a novel high performance SPI-based adhesive. The effects of TPU and KH-560 addition on the performance of the adhesive and the resultant plywood were investigated. Three-ply plywood was fabricated, and its dry/wet shear strength was tested according to China National Standards (GB/T 9846.3-2004). The functional groups, fracture morphology, thermal behavior, residual rate after hydrolyzing, fracture section and crack observations of the resultant adhesives were examined.

## 2. Materials and Methods

### 2.1. Materials

Soy protein isolate (SPI) (≧95% protein content) was obtained from Yuwang Ecological Food Industry Co., Ltd. (Shandong, China) and milled to 250 mesh flour. Commercial thermoplastic polyurethane elastomer (TPU) andγ-(2,3-epoxypropoxy)propyltrimethoxysilane (KH-560) were purchased from Lanyi Chemical Co., Ltd. (Beijing, China). The Cathay poplar veneers with a moisture content of 8.0% and dimensions of 400 mm × 400 mm × 1.5 mm were obtained from Tianjin Hai Yi Wood Industry Co., Ltd. (Tianjin, China). The other chemical reactants were analytical grade and obtained from Beijing Chemical Reagents Co. Ltd. (Beijing, China).

### 2.2. Preparation of the TGA

**Figure 1:**
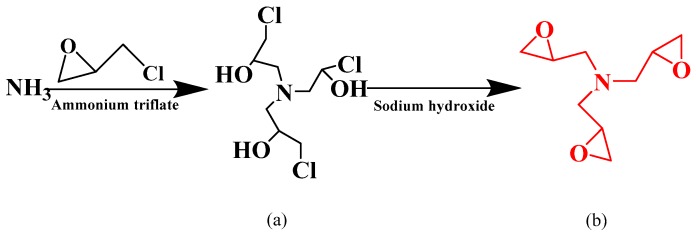
The synthesis procedure for triglycidylamine and its chemical structure. (**a**) tris(3-chloro-2-hydroxypropyl) amine; (**b**) TGA.

TGA was synthesized according to a previous report [15], the reaction pathway is illustrated in Figure 1. Epichlorohydrin and aqueous ammonia with a mole ratio 5:1 were placed into a three-necked flask (Beijing Lanyi Chemical Co., Ltd., Beijing, China) equipped with a condenser (Beijing Yi Su Bo Gu Biotechnology Co., Ltd., Beijing, China) and stirrer (Beijing Yi Su Bo Gu Biotechnology Co., Ltd.). The mixture was stirred continuously with a rate of 800 rpm. Ammonium triflate was used to catalyze the reaction at 23 °C for 48 h, then 35 °C for 3 h. The residual epichlorohydrin and ammonium hydroxide were removed by vacuum distillation, and the result was a colorless syrup consisted mostly of tris(3-chloro-2-hydroxypropyl) amine (Figure 1a). An excess of sodium hydroxide solution (50%) was added for the epoxy-ring closure reaction at 20 °C for 2 h. Because the reaction was highly exothermal, an external ice-water cooling circulator (Beijing Yi Su Bo Gu Biotechnology Co., Ltd.) was required to hold the temperature. The precipitate of sodium chloride was filtered off, and the residue was vacuum distilled to obtain pure viscous TGA (Figure 1b). 

### 2.3. Preparation of the Different Adhesives

SPI adhesive (adhesive A): SPI (15 g) was added to distilled water (85 g) and stirred for 10 min at 25 °C.

SPI/TGA adhesive (adhesive B): The SPI adhesive (100 g) and TGA (2 g) were mixed and stirred for 10 min at 25 °C.

SPI/TGA/TPU adhesive (adhesive C): TPU (7 g) was added to the SPI/TGA (100 g) adhesive and stirred for 10 min at 25 °C to form a homogeneous system.

SPI/TGA/KH-560 adhesive (adhesive D): SPI/TGA (100 g) and KH-560 (1 g) were mixed and stirred for 10 min at 25 °C.

SPI/TGA/KH-560/TPU adhesive (adhesive E): TPU (7 g) was added to the SPI/TGA/KH-560 (100 g) adhesive and stirred for 10 min at 25 °C to form a homogeneous system.

### 2.4. Preparation of Three-Ply Plywood Samples

Three-ply plywood samples were prepared according to following conditions. The different adhesives were applied to one side of the poplar veneer (400 mm × 400 mm × 1.5 mm) at a spread rate of 250 g/m^2^. Uncoated veneers were stacked between two adhesive-coated veneers and the grain of the two adjacent veneers was perpendicular to each other. The conditions of the hot-pressing processes of the assembled veneers are shown in Table 1. The temperature of hot pressing is 130 °C. The coated three-layer plywood was placed on a thermocompressor (Foshan Zhongke Machinery Co., Ltd., Foshan, China). Firstly, the pressure was raised to 0.8 MPa, and held for 8 min. Then, the pressure was reduced to 0.4 MPa, and held for 4 min. Finally, the pressure was decompressed to 0 MPa, and the hot pressing work completed. Six plywood samples were obtained for each formulation of adhesive. In this research, we extended the hot press time to 12 min for the TPU, making sure the TPU completely melted and reacted with the adhesive system.

### 2.5. Adhesive Strength Measurement

**Figure 2:**
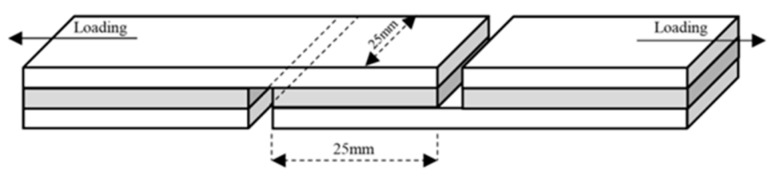
The schematic diagram of the sample preparation and the force loading.

The shear strength of plywood was determined according to the procedure described in China National Standard for Type II plywood (GB/T 17657-2013) [16]. In this study, we measured dry shear strength and wet shear strength. The abovementioned three-ply plywood were stored at atmospheric environment for 24 h, and then cut into specimens with dimensions of 100 mm × 25 mm (25 mm × 25 mm of gluing area). The schematic diagram of the sample preparation and the force loading is shown in Figure 2.

Twelve plywood specimens cut from two plywood panels were used to determine dry/wet shear strength. The dry shear strength of the six plywood specimens was determined using a common tensile machine operating at a speed of 10.0 mm/min. The force required to break the glued specimen was recorded. The shear strength was evaluated using Equation (1). Meanwhile, the rest of the specimens were immersed in water at 63 °C for 3 h and then measured under the same testing conditions. The acquired shear strength was an average value of twelve specimens and the standard deviation was given.

(1) $$\mathrm{Dry}/\mathrm{wet}\;\mathrm{shear}\;\mathrm{strength}\;\left(\mathrm{MPa}\right)\;=\;\frac{\mathrm{Force}\left(N\right)}{\mathrm{Gluing}\;\mathrm{area}\left(m^{2}\right)}$$

### 2.6. Determination of the Non Hydrolysable Residue

Each adhesive sample was cured in an oven (Beijing Lanyi Chemical Co., Ltd.) at 130 °C, and then ground into 100-mesh powder (0.15 mm) by a ceramic mortar (Beijing Lanyi Chemical Co., Ltd.). To evaluate the mass loss, the powder was wrapped up using filter paper (Beijing Lanyi Chemical Co., Ltd.) and placed in an oven at 103 ± 2 °C until a constant weight was obtained, then the weight of the filter paper (n) and the sample (m) were recorded respectively. Later, the samples were soaked in a glass (Beijing Lanyi Chemical Co., Ltd.) with distilled water. After hydrolyzing for 24 h, the samples were placed in an oven at 103 ± 2 °C until a constant weight was obtained (M). The residual proportion was determined due to Equation (2). The average value of the residual proportion was calculated from six parallel samples.

(2) $$\mathrm{Residual}\;\mathrm{rate}\;\left(\%\right)\;=\;\frac{\left(M-n\right)\left(g\right)}{\left(m-n\right)\left(g\right)}\times 100\%$$

### 2.7. Fourier Transform Infrared (FTIR) Spectroscopy

Each adhesive sample was placed in an oven at 130 °C to cure completely, then ground into 200-mesh powder. Fourier transform infrared (FTIR) spectra were recorded on a Nicolet 6700 spectrometer (Nicolet Instrument Corporation, Madison, WI, USA) using potassium bromide (KBr) crystal mixed with the adhesive powder at a mass ratio of 70/1 in the range of 4000–500 cm^−1^ with a 4 cm^−1^ resolution and 32 scans.

### 2.8. Scanning Electron Microscopy (SEM)

The adhesive samples were completely cured in an oven at 130 °C. Then, the samples were placed into a desiccator (Beijing Lanyi Chemical Co., Ltd.) for 2 days before being examined. The cured adhesives were artificially fractured, and the fracture surfaces were tested. The fracture surfaces of cured adhesive were sputter-coated with gold for 1 min to ensure sufficient conductivity and then observed under a Hitachi S-4800 emission scanning electron microscope (Hitachi Scientific Instruments, Tokyo, Japan) under 1000 and 5000 magnification.

### 2.9. Thermal Stability Measurement

The thermal degradation behavior of the cured samples was tested by using TA Q50 (Waters Company, New Castle, DE, USA). About 4–5 mg of powdered adhesive sample with 200 meshes was weighted in a platinum cup and scanned from room temperature to 610 °C at a heating rate of 10 °C /min. The weight changes were recorded in a nitrogen environment.

### 2.10. Cracks Observation

For visual evaluation of the toughness of cured adhesive, the adhesives were uniformly coated on 1.1 mm glass slides (Beijing Lanyi Chemical Co., Ltd.) with a rate of 200 g/m^2^, and then placed in an oven at a temperature of 130 ± 2 °C for 2 h. After adhesive curing, the coated glass slides were placed in a desiccator for 30 min. The cooled glass slides were photographed using a Digital Single Lens Reflex (Canon 6D Mark II).

## 3. Results

### 3.1. FTIR Analysis

**Figure 3:**
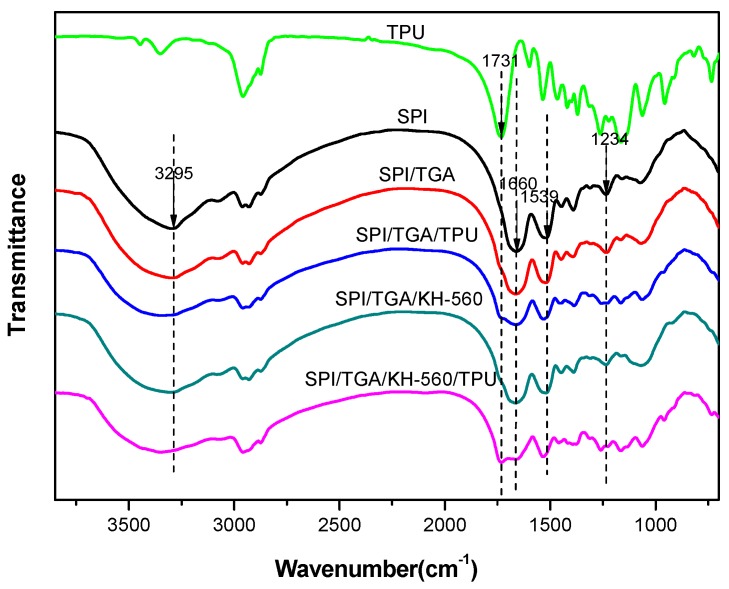
Fourier transform infrared (FTIR) spectra of the thermoplastic polyurethane elastomer (TPU) and different cured adhesives.

Figure 3 shows the FTIR spectra of the TPU and the different cured protein-based adhesives. The SPI adhesive spectra exhibited the relevant peaks at 1660, 1539 and 1234 cm^−1^, which are characteristic of amide I (C=O stretching), amide II (N–H bending), and amide III (C–N and N–H stretching), respectively [17]. Peaks corresponding to the stretching vibration of COO– and C–O of hydroxyl groups bonded to carbon atoms were observed at 1396 and 1073 cm^−1^ respectively. The peak observed at approximately 2930 cm^−1^ was attributed to symmetric and asymmetric stretching vibrations of the –CH_2_ groups in the different adhesives. The peak observed at approximately 3295 cm^−1^ corresponded to free and bound N–H and O–H groups, which are the functional groups forming hydrogen bonds with the carbonyl group of the peptide linkage in the protein. The peak observed at 1731 cm^−1^ in the spectra of TPU corresponded to characteristic peak of ester linkage.

**Figure 4:**
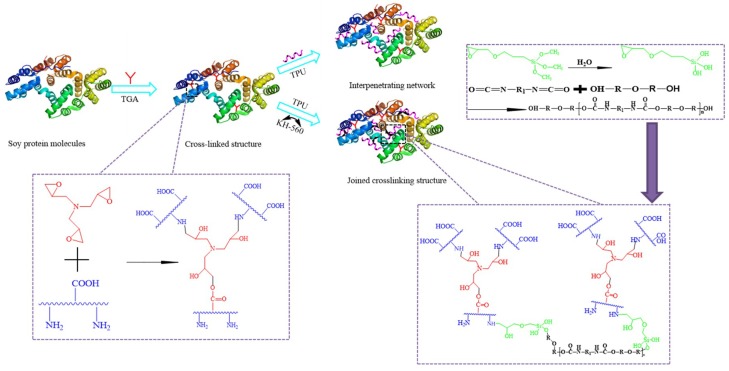
The reaction scheme of the adhesive.

After introducing TGA, the spectra of –COOH reduced and the amid I/II moved from 1661.9 to 1664.4 cm^−1^ (blue shift), indicating the epoxy group in TGA reacted with the active groups (–NH_2_, –OH) in the protein and formed a cross-linking structure, which benefited for the water resistance of the adhesive improvement. Adding KH560 in the SPI/TGA adhesive, no obvious differences was observed in the spectra of SPI/TGA/KH-560 adhesive, indicating a similar reaction happened after using KH-560 in the adhesive formulation. The epoxy group in KH560 reacts (–NH_2_, –OH) in protein, while the siloxane group forms a strong bond with the –OH in protein or wood. After introducing TPU, a minor peak was observed at 1731 cm^−1^, indicating the TPU was well distributed with the SPI based adhesive system. No obvious change of SPI/TGA/TPU adhesive spectra was observed compared with that of SPI/TGA adhesive, indicating the TPU was only physically combined with SPI/TGA adhesive to form an interpenetration structure. After KH-560 adding into SPI/TGA/TPU adhesive, the peak at 1731 (ester linkage) and 1256 cm^−1^ (C–N) of SPI/TGA/TPU/KH-560 adhesive increased, indicating KH-560 linked TPU with the siloxane group and protein with epoxy group to form a joined crosslinking structure. The reaction scheme is shown in Figure 4.

### 3.2. Non Hydrolysable Residue of Different Cured Adhesives

**Figure 5:**
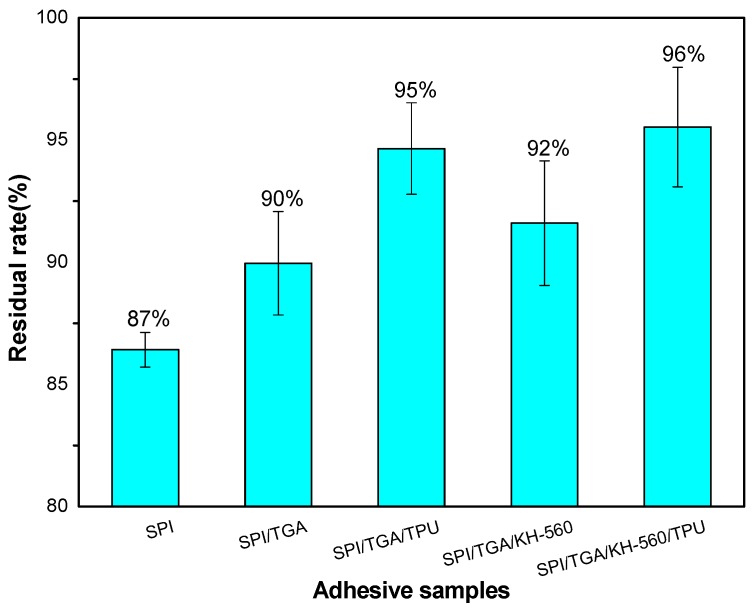
Non hydrolysable portion of different cured adhesives.

The hydrolytic stability of the adhesive was found by the determination of the non-hydrolyzable portion after hydrolysis at certain conditions, as described in Section 2.6. Figure 5 shows the residual rate of different cured adhesives. Because soy protein isolate contains many polar groups, it has good water absorption. So, the residual rate of the SPI adhesive was 87%, which was the lowest, indicating that the SPI adhesive had the lowest cross-linking degree among all the adhesives. After introducing 2 g TGA, the non-hydrolyzable portion of the adhesive increased by 4% compared with the SPI adhesive, owing to the cross-linking structures formed through the reaction between TGA and SPI. This result was in agreement with other studies based on the effects of use of an epoxy cross-linking agent on the adhesive [18]. Increase in the non-hydrolyzable portion is seen as absolute increase, as shown in Figure 5. This cross-linking structure prevents moisture intrusion and improved the hydrolytic stability [19]. When TPU were added to the SPI/TGA adhesive, the non-hydrolysable portion of the adhesive further increased by 5% compared to the SPI/TGA adhesive. From analysis of Figure 3, no chemical reaction was observed between TPU and SPI/TGA, indicating TPU physically mixed with SPI/TGA adhesive. During the hot press process, TPU melted and combined with the SPI/TGA adhesive to form an interpenetrated structure. This interpenetrated structure improved the water resistance of the adhesive due to the following reasons: (1) TPU was nonpolar material which could prevent water intrusion; (2) the formed interpenetrated structure wrapped protein molecule inside of the adhesive, which further improves the water resistance of the adhesive; and (3) TPU, as an elastomeric material, improved the toughness of the adhesive, which balanced the internal stress of the resultant plywood and improved the bond performance of the adhesive. When adding KH560 only into SPI/TGA, the non-hydrolyzable portion of the adhesive slightly increased by 2% comparing with the SPI/TGA adhesive. KH-560 has epoxy groups which reacts with active groups on the protein and further increases the crosslink density of the resulting adhesive. When using TPU and KH-560 together in the adhesive formulation, the residual rate further increased to 96%, which is 6% higher than that of SPI/TGA and 0.9% higher than that of SPI/TGA/TPU adhesive. This improvement is because KH-560 acting as a bridge connects with protein molecule and TPU to form a joined crosslinking structure. In addition, KH-560 also improved the interfacial force between adhesive and wood, which benefited to the bond performance of the resultant plywood.

### 3.3. Thermal Stability Analysis

**Figure 6:**
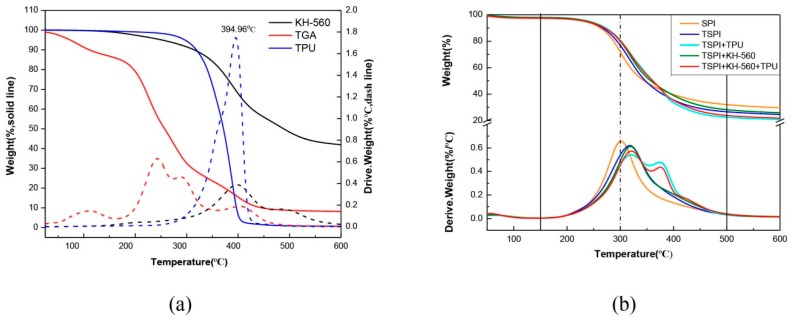
(**a**) TG (Thermogravimetry) and DTG (Derivative Thermogravimetric) curves of TPU, triglycidylamine (TGA), γ-(2,3-epoxypropoxy) propyltrimethoxysilane (KH-560), and (**b**) TG and DTG curves of the cured different adhesives.

The thermal degradation processes of TPU, TGA, KH-560, and the cured different adhesives are shown in Figure 6. The main thermal degradation process of SPI adhesive took place in the temperature ranged from 150 to 500 °C. The minor weight loss of the adhesive sample before 150 °C was owing to the evaporation of residual moisture [20]. In this process, no degradation of soy protein was found and the weight loss was less than 10%. Further increasing the temperature caused the degradation of soy protein involving separation of intermolecular structures and cleavage of the covalent bonding between the peptide bonds of amino acid residues. In SPI nearly 26% weight loss at 300 °C was attributed to the decomposition of the covalent bond cleavage between amino acid residues and peptide bonds. After introducing the TGA and KH-560/TGA, the weight loss of the adhesives decreased to 21% and 18%, respectively, indicating that the SPI/TGA and SPI/TGA/KH-560 adhesives had better thermal stability compared with the SPI adhesive. This is due to the crosslinking reaction between SPI and TGA or KH-560 to form a dense structure, which presented a better thermal stability. When TPU was introduced to the adhesive formulation, the weight loss of the SPI/TGA/TPU and SPI/TGA/KH-560/TPU adhesives further decreased to 17%, the addition of TPU, hence, showed only small effect in improvement of thermal stability.

From the DTG curve of SPI adhesive, a peak was observed at 300 °C, which was attributed to soy protein backbone peptide bonds degradation and produced various gases, such as CO, CO_2_, NH_3_, and H_2_S [21]. After introducing TGA and TGA/KH-560, this peak temperature increased from 300 °C to approx. 320 °C for all other adhesives due to the formation of more stable chemical bonds by the cross-linking reaction between TGA/KH-560 and soy protein molecules. After TPU introducing in the SPI/TGA adhesive, a new peak appeared at 390 °C, which was attributed to the degradation of TPU (Figure 6a). This also indicated no chemical reaction happened between TPU and SPI/TGA. The TPU combined with SPI/TGA to form a physically interpenetrating network. After incorporation of KH560 into the SPI/TGA/TPU adhesive, this peak was reduced, indicating that KH-560 linked SPI/TGA and TPU to form a joined crosslinking system, which would further increasing the performance of the resultant adhesive.

### 3.4. SEM Analysis

**Figure 7:**
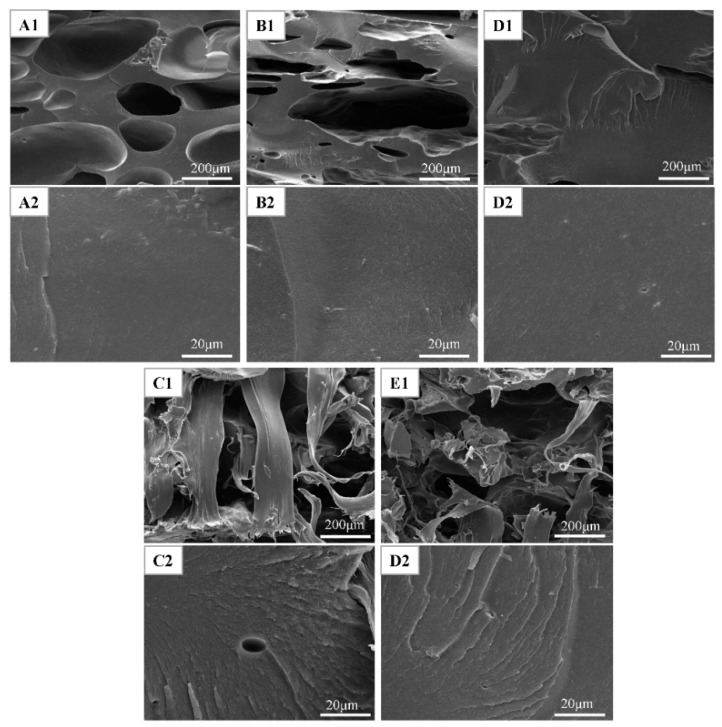
Fracture surface micrographs of the cured adhesive (**A1**,**A2**) (Soy protein isolate (SPI)), (**B1**,**B2**) (SPI/TGA), (**C1**,**C2**) (SPI/TGA/TPU), (**D1**,**D2**) (SPI/TGA/KH-560), (**E1**,**E2**) (SPI/TGA/KH-560/TPU).

The fracture surface micrographs of cured adhesives are shown in Figure 7A1–E1 shows the cross-sectional micrograph of cured adhesives under 100 magnification. A loose fracture surface with cracks was observed in the SPI adhesive, which revealed the inherent brittle and the cohesive failure caused by the evaporation of a large quantity of water (A1). These cracks provide a channel for moisture attack and caused the low water resistance of the adhesive [22]. The inherent brittleness of SPI adhesive could be aggravated by the cross-linking reaction, because the cured TGA (an epoxy-based cross-linker) is a brittle system. So, after TGA and TGA/KH-560 were added into the SPI adhesive, the cracks were fewer and the fracture surface became compact (see B1 and D1), suggesting that TGA and KH-560 cross-linking increased the cohesion of adhesive and simultaneously embrittled the cured system. When TPU was introduced into SPI/TGA adhesive, a wiredrawing phenomenon was observed in the fracture section of C1, suggesting TPU physically combined with the SPI/TGA adhesive. After adding KH-560, this wiredrawing decreased and more thin wiredrawing was observed, indicating the consistency between TPU and SPI/TGA was improved. Figure 7A2–E2 shows SEM images of different adhesives under 1000 magnification. It is noted that a smooth surface was obtained in the SPI adhesive and small cracks and holes were observed in the adhesive layer, indicating the SPI adhesive was a brittle system (A2). With the addition of TGA and KH-560, the adhesive layer of SPI/TGA and SPI/TGA/KH-560 adhesive became compact and had larger cracks. A denser structure was formed and the section presented a brittle fracture surface, which was a direct result of the cross-linking reaction that occurred between the TGA, KH-560 and the protein molecules in the SPI adhesive (see Figure 7B2,D2). By contrast, introduction of the TPU into the SPI/TGA adhesive resulted in a ductile fracture. It can be inferred that the addition of TPU improved the toughness of the adhesive by forming an interpenetrating network. Adding KH-560 in the SPI/TGA/TPU adhesive, a uniform ductile fracture section was observed, indicating the toughness of the adhesive further improved compared with TPU/SPI/TGA adhesive. The increase of adhesive toughness is beneficial to balance the interior force of the bonded products and improve their mechanical properties [23].

### 3.5. Cracks Observation of Cured Adhesive

**Figure 8:**
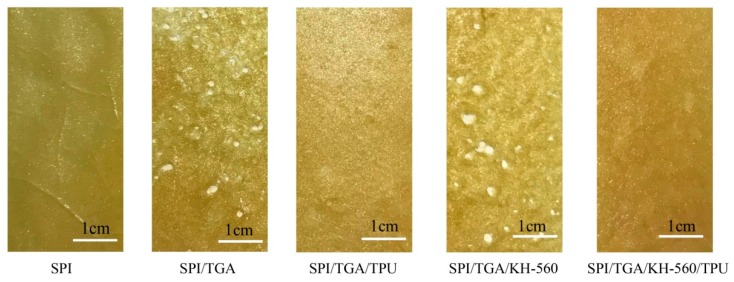
The cracks observation of the cured adhesive.

Figure 8 shows the cracks observed in the cured adhesives. Small cracks and holes were observed in the SPI adhesive layer, indicating that the SPI adhesive was a brittle system. After cross-linking by TGA or TGA/KH-560, the adhesive layer of SPI/TGA and SPI/TGA/KH-560 adhesive became compact and had larger cracks, which revealed the aggravated brittleness of the cured adhesive system, suggesting that the inherent brittleness of the SPI adhesive was deteriorated by forming a cross-linking structure. When introducing TPU, no cracks in the adhesive layer were observed and the surface became homogeneous and more compact than the SPI/TGA adhesive, suggesting TPU improved the toughness of the adhesive by forming an interpenetrating network. Moreover, the homogeneity and compactness was further increased with KH-560 introduction, indicating the toughness of the adhesive further improved by forming a joined crosslinking structure.

### 3.6. Shear Strength Measurement

**Figure 9:**
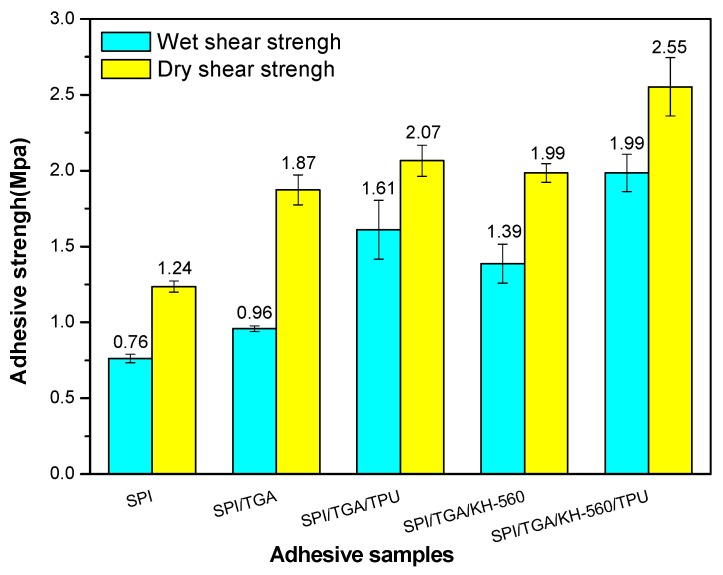
Dry and wet shear strength of the plywood of the cured adhesive.

Figure 9 shows the dry and wet shear strength of the resultant plywood bonded by the different adhesives. Because the hot pressing temperature and time are higher than previous studies [24], the SPI adhesive has reached 0.76 MPa, which meets the interior-use plywood requirement according to China National Standard (GB/T 9846.3-2004). The bond performance of SPI adhesive was primarily based on intermolecular hydrogen bonds and mechanical locking force, which presented the lowest bond performance among all adhesive samples. The SPI adhesive exhibited a low dry and wet shear strength of 1.24 and 0.76 MPa respectively. When 2% TGA was added to the SPI adhesive, the dry and wet shear strength increased by 51% and 26%, respectively. This result was in agreement with residual rate analysis. The epoxy groups in the cross linker reacted with active groups in the soy protein molecules to form a crosslinking structure (Figure 4), which prevented moisture intrusion and improved the water resistance of its resultant plywood. The wet shear strength improvement was not significant, which was attributed to the low addition of TGA.

With the addition of TPU, the dry and wet shear strength of plywood bonded by SPI/TGA/TPU adhesive increased by 11% and 68%, respectively, compared with that of SPI/TGA adhesive. TPU was distributed homogeneously in the adhesive system and formed an interpenetration network, which greatly improved the toughness of the adhesive, resulting in the improvement of the adhesive mechanical performance. The improvement of the dry bond strength was not significant compared with that of wet shear strength, which was attributed to the fact that the TPU and SPI/TGA adhesive physically penetrated each other and no chemical bond formed. When KH-560 was added into the SPI/TGA/TPU adhesive, the dry and wet shear strength reached to 2.55 and 1.99 MPa respectively, increasing by 36% and 107% compared with that of the SPI/TGA adhesive. The dry and wet shear strength of the plywood increased by 23% and 24% when compared with that of SPI/TGA/TPU adhesive. This indicated the formation of a joined crosslinking structure in the adhesive, further improving the performance of the adhesive.

## 4. Conclusions

In this work, an elastomeric material, TPU, was employed to modify the adhesive, as well as to improve the bond performance of a SPI-based adhesive. Introducing TGA and KH-560 as a cross-linker reacted with the functions of the soy protein and effectively increased the water resistance of the soy protein isolate adhesive. The wet shear strength of plywood bonded by SPI/TGA and SPI/TGA/KH-560 adhesive was 0.96 and 1.39 MPa, improving by 26% and 83% compared to plywood bonded by SPI adhesive. When TPU was added into the SPI/TGA adhesive, the dry and wet shear strength of the plywood bonded by the resultant adhesive increased by 67%, to 2.07 MPa, and 112%, to 1.61 MPa, respectively, compared with that of the SPI adhesive. Also, the residual rate of the SPI/TGA/TPU adhesive increased by 9% compared with that of SPI adhesive. This improvement was attributed to the TPU physically combining with SPI/TGA adhesive to form an interpenetration network: (1) TPU was nonpolar material which prevented water intrusion; (2) this formed interpenetration network wrapped protein molecule inside of the adhesive which further improved the water resistance of the adhesive; (3) TPU, as an elastomeric material, improved the toughness of the adhesive, which balanced the internal stress of the resultant plywood which improved the bond performance of the adhesive. When KH560 was introduced to the SPI/TGA/TPU adhesive, the dry bond strength of plywood bonded by SPI/TGA/TPU/KH560 adhesive increased by 23%, to 2.55 MPa, while the wet shear strength increased by 24%, to 1.99 MPa, compared with that of SPI/TGA/TPU adhesive. KH-560 acts as a bridge to connect TPU and SPI/TGA, forming a joined crosslinking network, which improved the thermostability and toughness of the adhesive, and at the same time created a uniform ductile fracture section of the adhesive. This joined network resulted in an improvement of the residual rate of the adhesive by 7% compared with that of SPI/TGA adhesive. The dry and wet shear strength of the plywood bonded by the SPI/TGA/TPU/KH-560 adhesive increased by 107% and 36% respectively when compared with that of the SPI/TGA adhesive.

## Figures and Tables

**Table 1:** Hot-pressing processes.

Process	Time/s	Pressure/MPa
Booster	5	0.8
Packing	485	0.8
Decompression	490	0.4
Packing	720	0.4
Decompression	725	0
Insulate	735	0
